# Genomic divergence, adaptation, and the genetic basis of quality traits in ancient walnut landraces and wild relatives

**DOI:** 10.1093/hr/uhag082

**Published:** 2026-03-31

**Authors:** Xiang Luo, Zhongzhong Guo, Qiang Jin, Zhenyang Shua, Honghua Zhang, Bin Wang, Chunhua Liu, Xin Chen, Haifang Hu, Shangqi Yu, Weiqiang Zhang, Wen Yao, Kai Ma, Rui Zhang

**Affiliations:** The National and Local Joint Engineering Laboratory of High Efficiency and Superior-Quality Cultivation and Fruit Deep Processing Technology of Characteristic Fruit Trees in South Xinjiang, Tarim University, Alar, Xinjiang 843300, China; Xinjiang Production and Construction Corps Southern Xinjiang Characteristic Fruit Technology Innovation Center, Tarim University, Alar, Xinjiang 843300, China; Institute of Fruit and Vegetable, Academy of Agricultural Sciences of Xinjiang Uyghur Autonomous Region, Urumqi, Xinjiang 830091, China; The National and Local Joint Engineering Laboratory of High Efficiency and Superior-Quality Cultivation and Fruit Deep Processing Technology of Characteristic Fruit Trees in South Xinjiang, Tarim University, Alar, Xinjiang 843300, China; Xinjiang Production and Construction Corps Southern Xinjiang Characteristic Fruit Technology Innovation Center, Tarim University, Alar, Xinjiang 843300, China; The National and Local Joint Engineering Laboratory of High Efficiency and Superior-Quality Cultivation and Fruit Deep Processing Technology of Characteristic Fruit Trees in South Xinjiang, Tarim University, Alar, Xinjiang 843300, China; Xinjiang Production and Construction Corps Southern Xinjiang Characteristic Fruit Technology Innovation Center, Tarim University, Alar, Xinjiang 843300, China; College of Life Sciences, Henan Agricultural University, Zhengzhou, Henan 450046, China; The National and Local Joint Engineering Laboratory of High Efficiency and Superior-Quality Cultivation and Fruit Deep Processing Technology of Characteristic Fruit Trees in South Xinjiang, Tarim University, Alar, Xinjiang 843300, China; Xinjiang Production and Construction Corps Southern Xinjiang Characteristic Fruit Technology Innovation Center, Tarim University, Alar, Xinjiang 843300, China; Institute of Fruit and Vegetable, Academy of Agricultural Sciences of Xinjiang Uyghur Autonomous Region, Urumqi, Xinjiang 830091, China; Aksu Regional Secondary Vocational and Technical School, Aksu, Xinjiang 843099, China; Walnut and Chestnut Nursery in Tai'an National Fruit Germplasm, Shandong Institute of Pomology, Tai'an, Shandong 271000, China; Xinjiang Academy of Forestry, Urumqi, Xinjiang 830000, China; The National and Local Joint Engineering Laboratory of High Efficiency and Superior-Quality Cultivation and Fruit Deep Processing Technology of Characteristic Fruit Trees in South Xinjiang, Tarim University, Alar, Xinjiang 843300, China; Xinjiang Production and Construction Corps Southern Xinjiang Characteristic Fruit Technology Innovation Center, Tarim University, Alar, Xinjiang 843300, China; Henan Scientific Research Platform Service Center, Zhengzhou, Henan 450003, China; College of Life Sciences, Henan Agricultural University, Zhengzhou, Henan 450046, China; Institute of Fruit and Vegetable, Academy of Agricultural Sciences of Xinjiang Uyghur Autonomous Region, Urumqi, Xinjiang 830091, China; The National and Local Joint Engineering Laboratory of High Efficiency and Superior-Quality Cultivation and Fruit Deep Processing Technology of Characteristic Fruit Trees in South Xinjiang, Tarim University, Alar, Xinjiang 843300, China; Xinjiang Production and Construction Corps Southern Xinjiang Characteristic Fruit Technology Innovation Center, Tarim University, Alar, Xinjiang 843300, China

## Abstract

Elucidating the genetic architecture of key quality traits and understanding population differentiation in walnut (*Juglans regia*) are critical for advancing molecular breeding and enhancing environmental adaptability. In this study, we performed whole-genome resequencing of 282 representative walnut accessions encompassing wild populations, ancient landraces, and modern cultivars. Walnuts in Xinjiang exhibit clear genetic differentiation and can be categorized into two main groups: wild walnuts from Yili and ancient landraces from Aksu, Kashi, and Hotan. The Yili region in northern Xinjiang, China, represents an independent center of walnut genetic diversity. In contrast, the ancient landraces and cultivated walnuts from other regions of Xinjiang are closely related to wild walnuts from the Middle East, suggesting a shared origin. Analysis of deleterious mutations uncovered contrasting accumulation patterns between Wild-Yili_China and Ancient Landrace-Xinjiang_China groups, likely shaped by demographic history and local environmental adaptation. Genome-wide association studies (GWAS) identified key loci and candidate genes, including *JruL2x*, *JrCRK26*, and *JrRHF2A*, that are associated with important quality traits. Furthermore, we functionally validated *JrCYP98A2*, which encodes a ferulate 5-hydroxylase, as a regulator of shell thickness. Together, these findings provide insights into walnut domestication and the genetic basis of quality trait variation, offering valuable genomic resources for future molecular breeding and genetic improvement of walnut.

## Introduction

The Persian walnut (*Juglans regia* L.), also known as the common walnut, is a diploid (2*n* = 2*x* = 32), monoecious, wind-pollinated species within the *Juglans* genus of the Juglandaceae family. This long-lived perennial is globally valued for its kernels, which are rich in protein and oil, making it an economically important crop [1]. In China, walnut cultivation dates back more than 7000 years, with the Xinjiang region serving as a historical center closely associated with early agricultural civilizations. The unique geographic and climatic conditions of Xinjiang have given rise to local walnut populations characterized by exceptional quality and environmental adaptability. To date, almost all elite walnut cultivars either originate from Xinjiang or possess the genetic background of Xinjiang walnuts. Additionally, Xinjiang is located in Central Asia and is considered one of the primary centers of walnut origin and domestication [2]. These factors highlight the significance of Xinjiang in walnut breeding and genetic diversity, providing valuable resources for future improvement efforts. However, systematic genetic research on Xinjiang walnut germplasm remains limited, and key genes and loci underlying locally adapted traits are largely unexplored. Furthermore, localized breeding efforts have progressed slowly, with a lack of elite cultivars, thereby constraining the high-quality development of the walnut industry.

Understanding the genomic diversity, population differentiation, and genetic basis of key agronomic traits is essential for accelerating crop improvement. The domestication and evolutionary history of cultivated walnut (*J. regia*) are particularly complex [1, 3], involving widespread ecological adaptation and extensive gene flow among distinct genotypic groups. Genomic analyses suggest that *J. regia* originated from an ancient hybridization between American and Asian walnut lineages during the late Pliocene, ~3.45 million years ago. It is widely accepted that *J. regia* survived the last glacial period in a series of refugia along the foothills of the Western Himalayas, including regions such as Kashmir, Tajikistan, Kyrgyzstan, and parts of South Asia. As the climate warmed after the glaciation, the heterogeneous topography and diverse ecological conditions of these areas facilitated the species’ adaptive divergence and shaped the present-day distribution of walnut populations. Additionally, human-mediated dispersal—facilitated by historical trade routes such as the Silk Road and the ancient Bronze Route—further contributed to the long-distance spread of walnuts [4].

Our recent research indicates that the core regions of *J. regia* genetic diversity are located in southwestern China and South Asia, particularly in areas adjacent to the Qinghai-Tibet Plateau and the Himalayas [2]. It is speculated that the uplift of the Himalayas played a pivotal role in shaping the present-day population structure and dynamics of Persian walnut. Based on early morphological classification, Chinese walnuts have traditionally been divided into four ecotypes: Xinjiang, Southwest China, Qinba, and North China [5]. Within Xinjiang, wild walnut populations are well preserved in natural reserves such as Gongliu County, while ancient landraces are especially abundant around the Tarim Basin. Despite these rich resources, there is still a limited understanding of the genetic diversity, evolutionary origins, and ecological adaptations of both wild and cultivated walnuts in these regions. In particular, how gene flow and evolutionary relationships between wild, landrace, and cultivated types have shaped walnut diversity and adaptation remains largely unexplored.

In recent years, the availability of chromosome-level reference genomes for various *Juglans* species has greatly facilitated the study of genes contributing to genetic differentiation and ecological adaptation [6]. Several candidate genes have been identified, including *JrGA20ox1* associated with drought tolerance [7]; a protein complex encoded by *JrPHL8*, *JrWRKY4*, and *JrSTH2L* that mediates anthracnose resistance [8]; and major-effect genes such as *JrFAD2* and *JrANR* that regulate linoleic acid content and nut endopleura color, respectively [9]. These findings underscore the potential of genomic approaches in identifying elite alleles valuable for breeding, particularly in underutilized germplasm like that of Xinjiang.

In this study, we performed whole-genome resequencing on a diverse panel of *J. regia* accessions, encompassing critical wild populations and ancient landraces from Xinjiang. First, we aimed to delineate the population structure and demographic history of Xinjiang walnuts and test the hypothesis of an independent domestication trajectory distinct from other centers of diversity. Next, we conducted GWAS to dissect the genetic architecture of key quality traits, such as tannin and oleic acid content, and to identify loci responsible for their variation. Finally, by identifying elite haplotypes and functionally validating a candidate gene for shell thickness, our study provides valuable genomic resources to guide future molecular breeding and enhance the genetic improvement of walnut.

## Results

### Genome resequencing of a comprehensive collection of 282 walnut accessions

**Figure 1:**
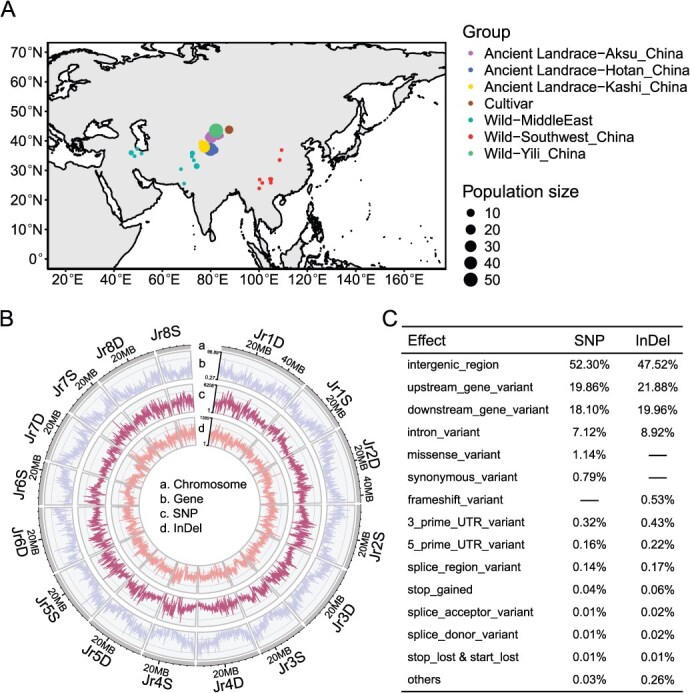
Geographic distribution and genome sequencing of 282 walnut accessions. (A) Geographic distribution of 282 walnut accessions. Different colors represent distinct genetic groups, and the size of the points indicates the number of accessions sampled at each location. (B) Circos plot depicting genomic features across the 16 walnut chromosomes. Tracks are as follows: (a) chromosome ideograms; (b) gene density; (c) SNP density; and (d) InDel density. Chromosomal coordinates are scaled in megabases (Mb). (C) Functional annotation of SNPs and InDels based on their predicted effects on gene structure and function.

We performed whole-genome resequencing on a comprehensive panel of 282 *Juglans* accessions, including 75 wild accessions collected from southwestern China, the Yili Prefecture in Xinjiang (China), and the Middle East; 193 ancient landraces from Xinjiang; 11 modern cultivars developed in Xinjiang; and 3 outgroup species (*Juglans nigra*, *Juglans hopeiensis*, and *Carya illinoinensis*) (Fig. 1A and Table S1). Among the 193 ancient landraces, 47 were sampled from Kashi, 80 from Hotan, and 66 from Aksu (Fig. 1A and Table S1). This sequencing effort generated short-read data at an average depth of ~20.1× (Table S1). On average, 96.31% of the cleaned reads were successfully aligned to the walnut reference genome, resulting in the identification of 11 883 354 single nucleotide polymorphisms (SNPs), with an average density of ~22 SNPs per kilobase (Kb) (Fig. 1B). In addition, 2 879 557 small insertions and deletions (InDels) were identified, with an average density of 5385 InDels per megabase (Fig. 1B). Among the identified SNPs, 0.79% were predicted to be synonymous (Fig. 1C). Notably, 0.53% of the InDels occurred in protein-coding regions and caused frameshift mutations, potentially exerting substantial effects on gene function and phenotypic variation (Fig. 1C).

### Phylogenetic and population structure analyses of 282 walnut accessions

**Figure 2:**
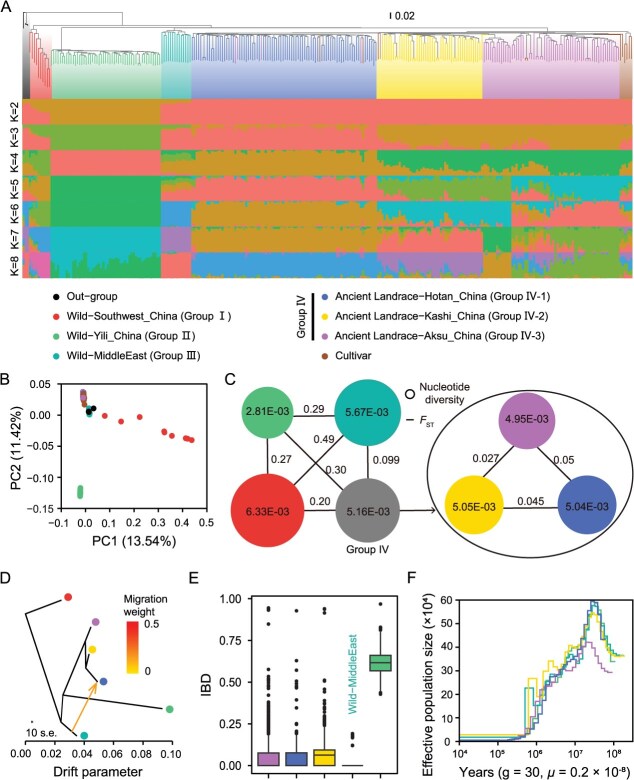
Population structure and genetic diversity of walnut accessions. (A) Population structure of 282 walnut accessions. The upper panel shows an NJ phylogenetic tree constructed using SNPs, with major clades highlighted by background colors corresponding to ancestral components identified by ADMIXTURE. The lower panel displays ancestry proportions from ADMIXTURE analysis for *K* = 2 to 8; each bar represents an individual, and different colors in these bars represent distinct ancestral components. Defined groups are indicated by colored labels below the ADMIXTURE plot. (B) PCA based on genome-wide SNPs. The first two principal components (PC1 and PC2), explaining 13.54% and 11.42% of total variance, respectively, are shown. Points are colored according to the defined population groups detailed in panel A. (C) Nucleotide diversity (*θ*_π_, shown inside circles) within walnut population groups and pairwise population differentiation (*F*_ST_, shown on connecting lines) among groups. Circle color corresponds to groups as defined in panel A. Subgroups of the ‘Ancient Landrace-Xinjiang_China’ cluster are shown as smaller circles. (D) Gene flow among walnut subpopulations inferred using TreeMix. (E) IBD sharing levels within selected groups: Wild-MiddleEast, Wild-Yili_China, Ancient Landrace-Kashi_China, Ancient Landrace-Aksu_China, and Ancient Landrace-Hotan_China. Boxplots illustrate IBD score distributions, with colors matching those in panel A. (F) Historical effective population size (*N_e_*) dynamics of five walnut groups inferred using the PSMC model. Colors correspond to panel A. The *x*-axis indicates time before present (in years), assuming a generation time g = 30 years and a mutation rate *μ* = 0.2 × 10^−8^ per site per generation. Convergence of *N_e_* curves around one million years ago indicates the most recent common ancestry of these *J. regia* groups.

To investigate the evolutionary relationships between Xinjiang walnuts and those from other regions, as well as the genetic differentiation within Xinjiang walnuts, we performed comprehensive phylogenetic and population structure analyses on the 282 walnut accessions using ~11.88 million SNPs. The analyses divided the walnut accessions into four distinct groups (Fig. 2A). Group I consisted of 10 wild accessions from Yunnan, Sichuan, Shanxi, and Guizhou provinces in southwestern China, designated as the Wild-Southwest_China group. Group II included all 51 wild accessions from the Yili Prefecture in Xinjiang, referred to as the Wild-Yili_China group. Group III was made up of 14 wild accessions from the Middle East, forming the Wild-MiddleEast group. Group IV, designated as the Ancient Landrace-Xinjiang_China population, comprised all 193 ancient landraces. Additionally, 11 modern cultivars were associated with this lineage: five were intermixed with the ancient landraces in the phylogenetic tree, while the remaining six formed a closely adjacent clade. Groups III and IV clustered as a sister clade, indicating a close evolutionary relationship. The phylogenetic analysis suggests that Groups III and IV exhibit a pattern of parallel evolution in relation to Group II. Furthermore, modern cultivars in Xinjiang appear to have originated from ancient landraces, with limited subsequent expansion of genetic diversity. This evolutionary pattern resembles that observed in other perennial fruit crops, such as apple, where modern cultivars descend from a narrow pool of ancestral genotypes [10].

Accessions within Group IV were further divided into three subclades: Group IV-1, Group IV-2, and Group IV-3, each exhibiting distinct geographical distribution (Fig. 1A). Group IV-1 primarily included landraces from Hotan, referred to as the Ancient Landrace-Hotan_China group. Group IV-2 predominantly consisted of accessions from Kashi, with the remainder from Aksu, collectively forming the Ancient Landrace-Kashi_China group. Most accessions from Aksu were clustered into Group IV-3, designated as the Ancient Landrace-Aksu_China group. However, we observed that some landraces from Aksu were intermixed with those from Kashi and Hotan in both phylogenetic tree and principal component analyses (PCAs) (Fig. 2A and B).

### Genomic diversity and linkage disequilibrium analysis of 282 walnut accessions

The whole-genome nucleotide diversity (θπ) across all walnut accessions was estimated to be 5.5 × 10^−3^ (Table S2), a level comparable to that observed in pear (5.5 × 10^−3^ [11]) and grapevine (5.1 × 10^−3^ [12]), but significantly higher than that in other perennial trees such as peach (1.5 × 10^−3^ [13]) and cassava (2.6 × 10^−3^ [14]). This relatively high level of nucleotide diversity suggests that Persian walnut has retained substantial genetic variation, likely due to its complex evolutionary history and broad geographical distribution [3]. Interestingly, wild walnut populations exhibited lower nucleotide diversity (4.87 × 10^−3^) than both cultivated accessions (5.28 × 10^−3^) and ancient landraces (5.16 × 10^−3^) (Fig. 2C and Table S2). This trend mirrors patterns observed in other perennial fruit trees, such as pear [11], but contrasts with those of major annual crops such as soybean [15] and rice [16]. The reduced diversity in wild walnuts may reflect historical bottlenecks and adaptation to harsh environments [3, 17]. In contrast, the relatively higher diversity in cultivated walnuts is likely attributable to frequent cross-pollination inherent to their outcrossing nature, as well as extensive gene flow facilitated by human activity.

Among wild populations, the Wild-Yili_China group exhibited the lowest nucleotide diversity (2.81 × 10^−3^), compared to the Wild-MiddleEast (5.67 × 10^−3^), ancient landrace (5.16 × 10^−3^), and Wild-Southwest_China (6.33 × 10^−3^) (Fig. 2C and Table S2). The reduced diversity in the Wild-Yili_China group likely reflects a narrow genetic base resulting from prolonged bottlenecks and frequent inbreeding within a geographically and ecologically restricted niche. This is further supported by Watterson’s estimator (θw), as well as by the relatively low number of SNPs and InDels within this group (Table S2). Moreover, the Wild-Yili_China group exhibited a notably slower decay of linkage disequilibrium (LD) compared to other subpopulations (Fig. S1). This slower LD decay is consistent with a history of limited genetic recombination and gene flow, likely driven by long-term geographic isolation and ecological constraints specific to the Yili region.

### Domestication and geographical differentiation of walnuts

To investigate genetic differentiation among walnut groups, we calculated pairwise fixation index (*F*_ST_) values. The highest differentiation (*F*_ST_ = 0.49) was observed between the Wild-MiddleEast and the Wild-Southwest_China groups (Fig. 2C). Moderate differentiation was detected between the Wild-Yili_China and Wild-MiddleEast groups (*F*_ST_ = 0.29), and between the Wild-Yili_China and Wild-Southwest_China groups (*F*_ST_ = 0.27) (Fig. 2C), supporting the hypothesis of multiple centers of genetic diversity for walnut, as suggested in previous studies [18–20]. The lowest differentiation (*F*_ST_ = 0.099) was found between the Wild-MiddleEast group and the Ancient Landrace-Xinjiang_China group (Fig. 2C), consistent with phylogenetic and structure analyses (Fig. 2A), suggesting that the Ancient Landrace-Xinjiang_China and the Wild-MiddleEast group likely share a common ancestry. The inferred gene flow from Wild-MiddleEast walnuts into the ancient landraces of Hotan further supports this genetic relationship (Fig. 2D).

To assess the extent of shared genomic segments, we performed identity-by-descent (IBD) analysis among different walnut groups (Fig. 2E). The Wild-Yili_China group exhibited higher within-group IBD values than both the Wild-MiddleEast group and the Ancient Landrace-Xinjiang_China group, indicating a history of prolonged inbreeding or restricted gene flow, and resulting in a more homogeneous genetic background within the Wild-Yili_China group.

Positive Tajima’s D values were detected across all six subpopulations, implying that these populations may have undergone balancing selection or experienced population contraction (Table S2). Furthermore, pairwise sequentially Markovian coalescent (PSMC) analysis revealed a marked decline in effective population size in all groups ~2 million years ago (Fig. 2F). This demographic contraction coincides with the Quaternary Ice Age in Asia, a period marked by dramatic climatic fluctuations that likely imposed strong selective pressures on walnut populations [20]. These historical events may have contributed to the genetic bottlenecks observed in some groups, particularly the Wild-Yili_China population, which also exhibits reduced nucleotide diversity.

### Adaptive genomic divergence between Wild-Yili_China and Ancient Landrace-Xinjiang_China groups

**Figure 3:**
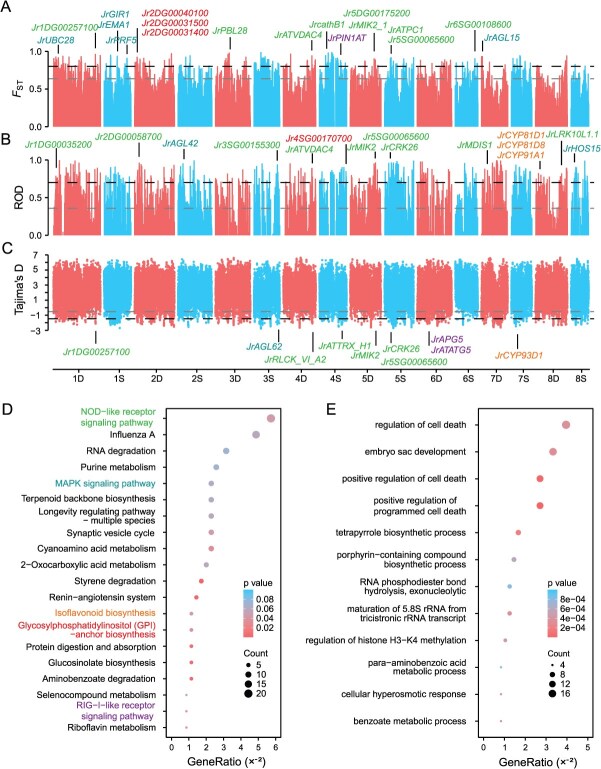
Genomic divergence between Wild-Yili_China and Ancient Landrace-Xinjiang_China populations. (A–C) Genome-wide distributions of fixation index (*F*_ST_) (A), ROD (B), and Tajima’s D (C) in 100-kb sliding windows, highlighting regions of high divergence between the Ancient Landrace-Xinjiang_China and Wild-Yili_China groups. Dashed black and gray lines indicate top 1% and 5% thresholds, respectively. Key candidate genes within these regions are labeled. (D–E) KEGG pathway (D) and GO (E) enrichment analysis of genes located in the highly divergent genomic regions. In both panels D and E, enriched terms are shown as dots, with dot size reflecting the number of genes and dot color indicating statistical significance (*P* value).

To identify genomic regions contributing to differentiation between Wild-Yili_China and Ancient Landrace-Xinjiang_China populations, we performed a genome-wide scan based on *F*_ST_, reduction of diversity (ROD), and Tajima’s D values (Fig. 3A–C). Genomic regions ranking in the top 1% of all three metrics were considered highly differentiated. This analysis identified 68 divergent genomic regions spanning 13.95 Mb (3.43%) of the walnut reference genome and encompassing 1217 predicted protein-coding genes (Tables S3 and S4).

Functional enrichment analysis of these 1217 genes revealed significant overrepresentation of the RIG-I-like receptor, NOD-like receptor, and MAPK signaling pathways (Fig. 3D), which are known to play pivotal roles in transducing cellular signals in response to biotic and abiotic stresses [21]. These pathways are crucial for the adaptation and survival of walnut trees under diverse environmental conditions.

Notably, four cytochrome P450 genes—Jr7SG00076700 (*JrCYP93D1*), Jr8DG00045500 (*JrCYP91A1*), Jr8DG00045400 (*JrCYP81D1*), and Jr8DG00045300 (*JrCYP81D8*)—were located within these highly divergent regions. These genes are involved in the biosynthesis of isoflavonoids, compounds known to mediate plant responses to drought stress [22]. Another cytochrome P450 gene, *JrCYP38*, has also been previously implicated in photosynthetic responses to drought stress in Persian walnut [23]. Additionally, four genes involved in the biosynthesis of glycosylphosphatidylinositol (GPI) anchors, known regulators of drought stress responses in poplar [24], were identified in divergent regions. Gene ontology (GO) enrichment analysis further indicated that the differentiated genes were primarily involved in biological processes such as the regulation of cell death, metabolic processes, and cellular responses to hyperosmotic stress (Fig. 3E).

Collectively, these findings suggest that environmental pressures—particularly drought—have been key drivers of the differentiation between wild walnuts in Yili and ancient landraces in other regions of Xinjiang. The identified candidate genes are likely contributors to local adaptation and may have facilitated walnut survival in distinct ecological zones across Xinjiang.

### Deleterious mutation analysis in Wild-Yili_China and Ancient Landrace-Xinjiang_China populations

**Figure 4:**
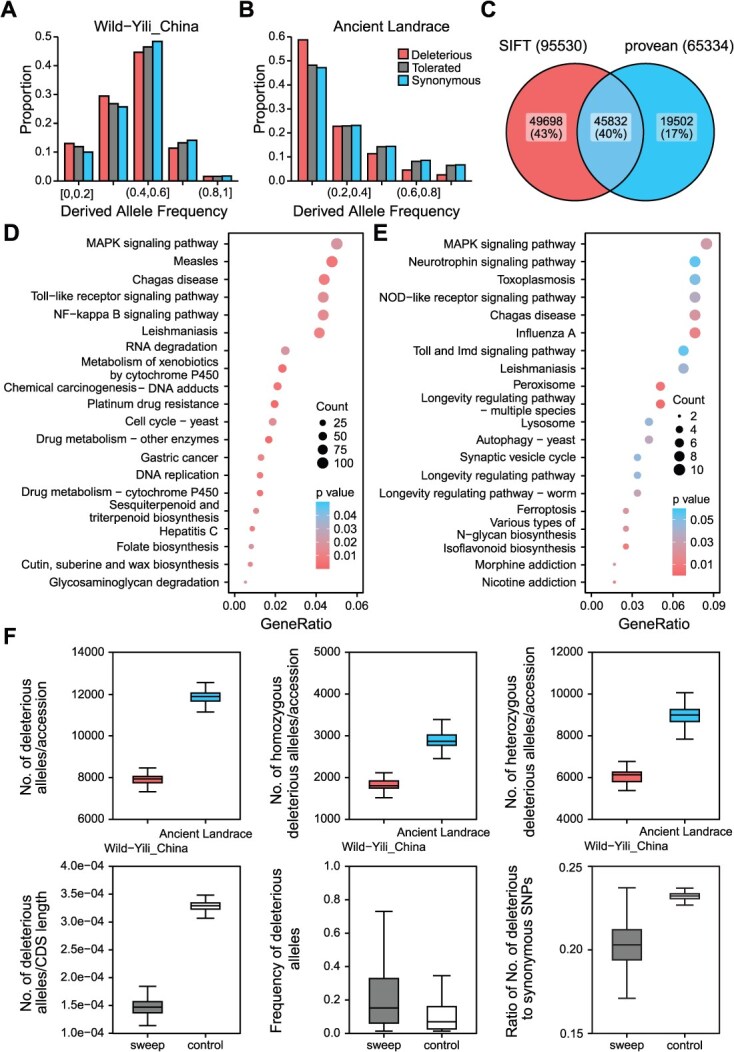
Deleterious mutations in the walnut population. (A–B) Site frequency spectra of nonsynonymous deleterious and synonymous mutations in the Wild-Yili_China (A) and Ancient Landrace-Xinjiang_China (B) populations. (C) Venn diagram showing the number of unique and shared nonsynonymous deleterious mutations predicted by SIFT and PROVEAN. (D–E) KEGG pathway enrichment analyses of genes harboring nonsynonymous deleterious mutations in Wild-Yili_China (D) and Ancient Landrace-Xinjiang_China (E), respectively. (F) Number and frequency of derived deleterious alleles in Wild-Yili_China and Ancient Landrace-Xinjiang_China. The top row shows the number of deleterious mutations per individual, including total (left), homozygous (middle), and heterozygous (right) counts. The bottom row presents a comparison between divergent genomic regions (labeled ‘sweep’) and the genomic background (‘control’), displaying the number of deleterious mutations normalized by CDS length (left), the frequency of deleterious alleles (middle), and the ratio of deleterious to synonymous variants per individual (right).

Deleterious mutations influence genetic differentiation among populations through selective purging or accumulation, particularly during demographic events such as population expansion and domestication. To assess the impact of these mutations on walnut genetic differentiation, we identified putative deleterious nonsynonymous mutations. The derived allele frequency spectrum of these putative deleterious mutations was significantly skewed toward lower frequencies compared to other variant classes (Fig. 4A–C), consistent with purifying selection acting against these variants—a pattern also observed in other species such as barley and soybean [25].

In total, we identified 34 902 deleterious SNPs (dSNPs) affecting 13 493 genes across the walnut population (Table S5). The Wild-Yili_China group harbored 11 701 dSNPs within 5559 genes, whereas the Ancient Landrace-Xinjiang_China group contained 33 271 dSNPs across 13 097 genes. On average, individuals from the Ancient Landrace-Xinjiang_China group carried 49.62% more derived deleterious variants than those from the Wild-Yili_China group, reflecting substantial divergence in their evolutionary trajectories. Notably, the majority of deleterious variants were present in heterozygous states in both populations, suggesting the persistence of recessive deleterious alleles shielded from selection, a phenomenon previously documented in grape [26].

A pronounced difference was observed in private deleterious mutations: 7934 genes carried private deleterious mutations in the Ancient Landrace-Xinjiang_China group, contrasting sharply with only 396 such genes in the Wild-Yili_China group (Table S5). Kyoto Encyclopedia of Genes and Genomes (KEGG) pathway enrichment analysis revealed distinct functional specializations. In the Wild-Yili_China group, private deleterious mutations were enriched in genes related to biotic and abiotic stress responses, including isoflavonoid biosynthesis, MAPK signaling, and NOD-like receptor signaling pathways (Fig. 4D). Conversely, in the Ancient Landrace-Xinjiang_China group, private deleterious mutations were enriched in metabolic pathways such as xenobiotics metabolism by cytochrome P450, cutin, suberine and wax biosynthesis, and sesquiterpenoid and triterpenoid biosynthesis (Fig. 4E).

We further analyzed the distribution of deleterious variants within genetically differentiated regions in Ancient Landrace-Xinjiang_China walnuts, comparing them to nondifferentiated regions. The probability of deleterious mutations occurring within differentiated regions was 4.30E−3, higher than the 3.63E−3 observed in nondifferentiated regions (Fig. 4F). Additionally, although selective sweep regions showed significantly higher frequencies of deleterious mutations (*P* < 0.01), they contained fewer deleterious mutations when normalized by region length (*P* < 0.01) and when compared to synonymous variants (*P* < 0.01) (Fig. 4F).

These findings suggest that domestication has profoundly reshaped the landscape of deleterious mutations in Ancient Landrace-Xinjiang_China walnuts. The accumulation of deleterious mutations, particularly in proximity to selected loci, is likely a consequence of genetic drift during domestication bottlenecks or hitchhiking of linked alleles during artificial selection—a phenomenon known as the ‘cost of domestication’, documented previously in crops such as maize [27]. In summary, the distinct patterns of deleterious variant accumulation and distribution between Wild-Yili_China and Ancient Landrace-Xinjiang_China walnuts reflect their divergent evolutionary histories, shaped by adaptation to unique environmental pressures and the intense selective forces of domestication [4].

### Identification of loci associated with tannin content in walnut

**Figure 5:**
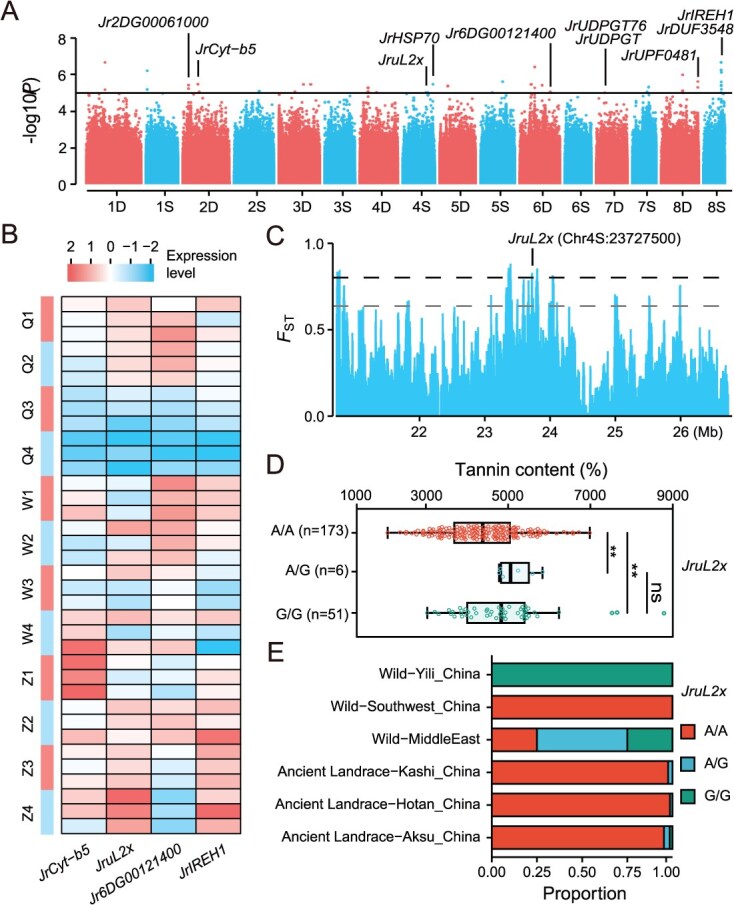
GWAS of tannin content in walnut. (A) Manhattan plot showing GWAS results for tannin content in walnut kernels. The horizontal solid line indicates the genome-wide significance threshold. Candidate genes located near significant SNPs are labeled. (B) Expression profiles of four candidate genes at four stages of kernel development in different genotypes. The tannin content of seed coat in ‘Q’ is lower than that of ‘W’ and ‘Z’. Samples were collected 70, 80, 90, and 100 days after flowering of the seed coat. Three biological replicates were performed for each sample. (C) Genetic differentiation (*F*_ST_) across a 10-Mb genomic region surrounding *JruL2x*, comparing the Ancient Landrace-Xinjiang_China and Wild-Yili_China groups. Dashed black and gray lines represent the top 1% and 5% *F*_ST_ thresholds, respectively. (D) Boxplots of tannin content in accessions harboring the three different genotypes at the lead SNP near *JruL2x*. Statistical significance was assessed using Wilcoxon rank-sum tests (^**^P < 0.01; NS, not significant). (E) Allele frequency distribution of the lead SNP across six walnut subpopulations.

Tannin content is a key determinant of walnut flavor and quality, with elevated levels often causing undesirable astringency. To elucidate the genetic basis of this important trait, we performed a GWAS on tannin content in 245 walnut accessions, which identified 27 significant loci across 14 chromosomes, pinpointing 10 genes as strong candidates involved in tannin biosynthesis (Fig. 5A and Fig. S2A, Table S6).

A particularly compelling association was detected on chromosome 7D, where the SNP Jr7D-7826622 highlighted two adjacent candidate genes: *Jr7DG00080700* (*JrUDPGT*) and *Jr7DG00080800* (*JrUDPGT76*) (Fig. 5A). Both encode UDP-glycosyltransferases (UGTs), key enzymes that catalyze the conversion of gallic acid to β-glucogallin—a committed step in hydrolysable tannin biosynthesis [28]. The functional significance of UGTs in modulating astringency is well documented in other species; for instance, they mediate the formation of bitter glycosides in tea leaves [29, 30]. Thus, the identification of these UGTs not only substantiates our GWAS findings but also reinforces their conserved role as key regulators of tannin metabolism across plants.

To further elucidate the gene expression patterns governing tannin accumulation, we integrated transcriptome data of the seed coat from various stages of kernel development for three genotypes. This analysis revealed four candidate genes—*Jr2DG00135200* (*JrCyt-b5*), *Jr4SG00132200* (*JruL2x*), *Jr6DG00121400*, and *Jr8SG00094800* (*JrIREH1*)—that were differentially expressed during kernel development, implicating them in tannin regulation (Fig. 5B). Among these, *JruL2x* on chromosome 4S was particularly noteworthy as it resides in a genomic region showing high divergence between the Wild-Yili_China and Ancient Landrace-Xinjiang_China groups (Fig. 5C). A significant SNP, Jr4S-23732595, located within the coding region of *JruL2x* (Jr4S:23727500–23732831), gives rise to three distinct genotypes: A/A, A/G, and G/G (Fig. 5D). Importantly, accessions carrying A/A exhibited significantly lower tannin content, corresponding to a more palatable flavor profile (Fig. 5D). The pronounced enrichment of the low-tannin A/A genotype in Ancient Landrace-Xinjiang_China strongly suggests that this allele has been a primary target of positive selection during walnut domestication (Fig. 5E).

### Identification of loci associated with oleic acid content in walnut

**Figure 6:**
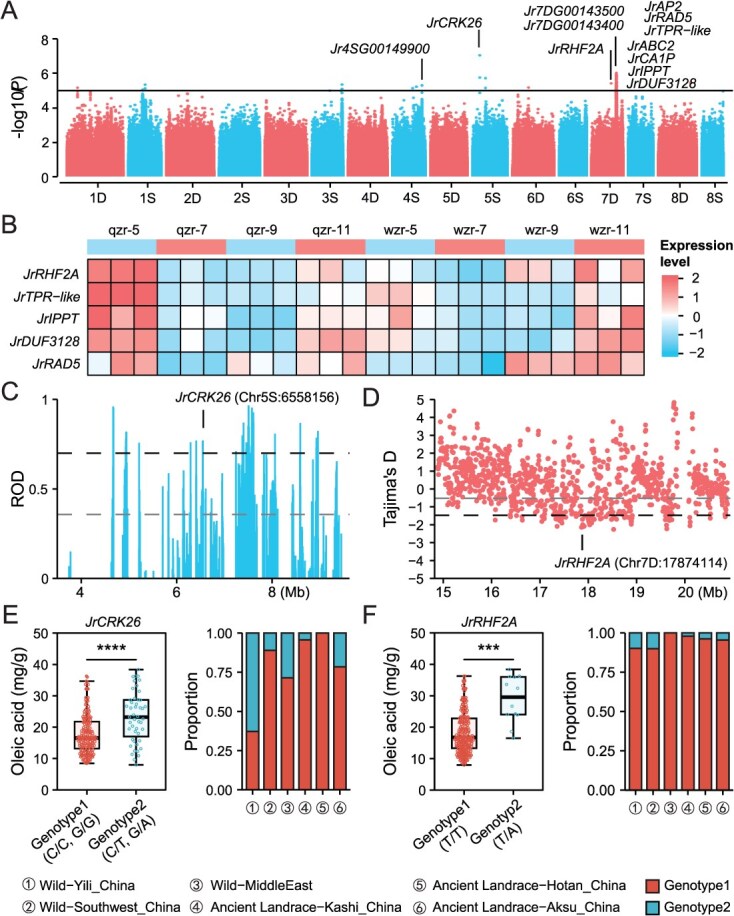
GWAS of oleic acid content in walnut. (A) Manhattan plot of GWAS results for oleic acid content. The horizontal solid line indicates the genome-wide significance threshold. Candidate genes located near significant SNPs are labeled. (B) Expression profiles of candidate genes at different stages of kernel development in different genotypes ‘qz’ and ‘wz’. The oleic acid content in ‘wz’ is higher than that in ‘qz’. Kernel samples were collected every 2 weeks from the initial formation stage (r-5) to the mature stage (r-11). (C, D) ROD (C) and Tajima’s D values (D) across a 10-Mb genomic region surrounding *JrCRK26*, comparing the Ancient Landrace-Xinjiang_China and Wild-Yili_China groups. Dashed black and gray lines indicate the top 1% and 5% thresholds, respectively. (E, F) Boxplots showing differences in oleic acid content among different genotypes at *JrCRK26* and *JrRHF2A*. Statistical significance was assessed using Wilcoxon rank-sum tests (^****^P < 0.0001; ^***^P < 0.001). Bar plots on the right display genotype frequencies across six walnut subpopulations.

Oleic acid, a monounsaturated fatty acid, plays a crucial role in determining the nutritional value and flavor profile of walnut kernels. In this study, GWAS of oleic acid content identified 14 significant loci distributed across eight chromosomes. Within these loci, we identified 12 genes potentially involved in oleic acid metabolism (Fig. 6A and Fig. S2B, Table S7). RNA-seq analysis further revealed five genes—*Jr7DG00126800* (*JrRHF2A*), *Jr7DG00143600* (*JrIPPT*), *Jr7DG00144200* (*JrTPR-like*), *Jr7DG00144900* (*JrDUF3128*), and *Jr7DG00145000* (*JrRAD5*)—that were differentially expressed during kernel development, suggesting their potential roles in regulating oleic acid accumulation (Fig. 6B). A significantly associated SNP, Jr7D-17885009, was located in the coding region of *JrRHF2A* (Chr 7D: 17 874 114–17 885 331), which encodes an E3 ubiquitin-protein ligase known to regulate various aspects of lipid biosynthesis during plant development [20]. This suggests that *JrRHF2A* likely plays a regulatory role in oleic acid accumulation in walnut kernels.

Another significant locus was identified on chromosome 7D (22 502 422–22 978 474 bp), encompassing nine protein-coding genes (Fig. 6A). Among these, *Jr7DG00143300* (*JrABC2*) encodes an ABC transporter, which facilitates the transport of long-chain fatty acyl-CoA from the cytoplasm into peroxisomes [31], a process essential for lipid homeostasis and oleic acid accumulation. Also within this region, *JrTPR-like* encodes a pentatricopeptide repeat (PPR)-containing protein (Fig. 6A), homologous to sesame PPR genes involved in regulating lipid biosynthesis during seed maturation [32]. These PPR genes have also been linked to seed coat coloration, suggesting a broader regulatory role in lipid metabolism including oleic acid biosynthesis.

Furthermore, two genes, *Jr5SG00065700* (*JrCRK26*; on chromosome 5S) and *JrRHF2A* (on chromosome 7D), resided within GWAS peaks and selective sweep regions (Fig. 6C and D). Two SNPs (Jr5S-6558295 and Jr5S-6558315) located within the coding sequence of *JrCRK26* (Jr5S:6558156–6562939) defined two distinct genotypes: Geno1 (C/C at Jr5S-6558295, G/G at Jr5S-6558315) and Geno2 (C/T at Jr5S-6558295, G/A at Jr5S-6558315). Notably, accessions carrying Geno2 exhibited significantly higher oleic acid content (Fig. 6E), indicating the potential of this genotype for improving the nutritional quality of walnut kernels. Interestingly, Geno2 occurred at a higher frequency in wild walnut accessions, particularly those from the Yili region, than in ancient landraces (Fig. 6E), highlighting Wild-Yili_China walnuts as valuable genetic resources for improving oleic acid content through targeted breeding. A similar trend was observed for *JrRHF2A*, where accessions carrying genotype T/A exhibited higher oleic acid levels than those with T/T. Moreover, the frequency of T/A was higher in the Wild-Yili_China and Wild-Southwest_China groups than in other groups (Fig. 6F), further underscoring the potential of Wild-Yili_China walnuts as donor lines for enhancing lipid quality traits in cultivated walnuts.

### Identification of loci associated with shell thickness in walnut

**Figure 7:**
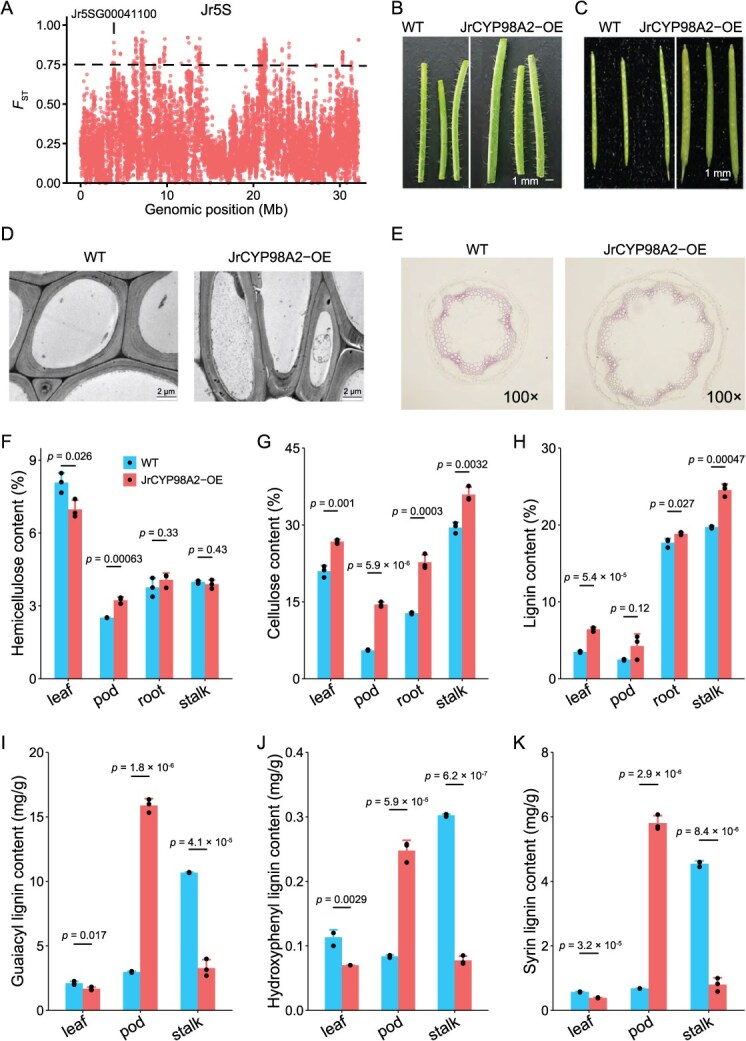
Identification and functional characterization of *JrCYP98A2* as a key regulator of lignification and shell thickness. (A) Population genomic analysis identifies *Jr5SG0041100* (*JrCYP98A2*) as a candidate gene for walnut shell thickness. *F*_ST_ values were calculated across the genome between thick- and thin-shelled walnut accessions. The dashed line indicates the threshold for candidate gene identification. (B, C) Stalks (B) and pod (C) of wild-type (WT) and *JrCYP98A2*-overexpressing (*JrCYP98A2*-OE) *A. thaliana* plants. Scale bar = 1 mm. (D) SEM images of stem cross-sections from WT and *JrCYP98A2*-OE *Arabidopsis* plants. Scale bar = 2 μm. (E) Phloroglucinol staining of stem cross-sections from WT and *JrCYP98A2*-OE *Arabidopsis* plants. Red coloration indicates lignified regions. Original magnification: 100×. (F–K) Comparison of hemicellulose content (F), cellulose content (G), total lignin content (H), guaiacyl lignin content (I), hydroxyphenyl lignin content (J), and syringyl lignin content (K) in various tissues of WT and *JrCYP98A2*-OE *Arabidopsis* plants. Data are presented as mean ± standard deviation (SD) of three biological replicates. *P* values were determined by Student’s *t* test.

Shell thickness is an important quality trait of walnut fruit, with thinner shells generally preferred. A previous GWAS study identified *JrPXC1* as a key regulator of secondary wall cellulose thickening in walnut shells [33]. In this study, we focused on walnut accessions exhibiting extreme shell thickness phenotypes and employed a population genetic differentiation approach based on *F*_ST_ to identify loci associated with shell thickness. This analysis revealed a candidate gene on chromosome 5, *Jr5SG00041100* (*JrCYP98A2*), a member of the F5H gene family (Fig. 7A). Expression profiling showed that *JrCYP98A2* was highly expressed in walnut fruit across different developmental stages, particularly in endocarp at 78 and 92 days postanthesis. In contrast, its expression was relatively low in the kernel, inner pericarp, and outer pericarp (Fig. S3). Subcellular localization assays indicated that the JrCYP98A2 protein is localized in the endoplasmic reticulum (Fig. S4).

To functionally characterize *JrCYP98A2*, we generated transgenic *Arabidopsis thaliana* plants overexpressing the gene (Fig. S5). Compared to wild-type Col-0, these transgenic lines exhibited varying degrees of stem and pod cortex thickening (Fig. 7B and C). Scanning electron microscopy (SEM) analysis of stem cell walls revealed partial detachment of the primary plasma membrane and thickening of the middle lamella in overexpressing lines, while the secondary wall appeared more distinct in wild-type plants (Fig. 7D). Toluidine blue staining of stem cross-sections revealed an increased number of lignified cells in transgenic plants; however, the proportion of the lignified cells relative to the total stem cross-sectional area was significantly reduced (Fig. 7E).

Biochemical assays measuring hemicellulose, cellulose, and total lignin contents showed no significant differences in hemicellulose levels in roots and stalks. However, hemicellulose content increased by 19.09% in pods and slightly decreased in leaves in transgenic plants compared to wild type (Fig. 7F). Cellulose content was elevated in leaves (27.31%), roots (41.68%), pods (61.08%), and stalks (17.90%) (Fig. 7G), while total lignin content increased by 45.56% in leaves and 19.64% in stalks (Fig. 7H). High-performance liquid chromatography analysis of lignin monomers showed that the levels of guaiacyl (G-type), hydroxyphenyl (H-type), and syringyl (S-type) lignin in leaves and stalks were significantly reduced in leaves and stalks of transgenic plants compared to wild type. In contrast, all three lignin monomer types were markedly increased in pods, by 81.23% (G-type), 66.40% (H-type), and 88.28% (S-type), respectively (Fig. 7I–K). Together, these findings suggest that *JrCYP98A2* plays a critical role in regulating lignin biosynthesis during pod development and is likely a key genetic factor regulating shell thickness in walnut.

## Discussion

### Large-scale population genomic data reveal the genetic differentiation of Xinjiang walnuts

Understanding the genetic differentiation of walnuts in Xinjiang is essential for guiding future genetic improvement and conservation efforts. Our analyses reveal that Xinjiang walnuts exhibit clear genetic differentiation, comprising two major populations: Wild-Yili_China and Ancient Landrace-Xinjiang_China (Fig. S6). Multiple lines of evidence—including phylogenetic analysis, population structure inference, fixation index (*F*_ST_) metrics, and PSMC modeling—support the hypothesis that the Yili region in Xinjiang, China, represents an independent center of genetic diversity for *J. regia*. Ancestry proportion analysis consistently revealed a unique ancestral component in wild walnuts from Yili, distinct from those found in wild walnut populations from Southwest China and the Middle East (Fig. 2A). The pronounced genetic divergence is further supported by the clear separation in PCA analysis and high *F*_ST_ values between the Wild-Yili_China and other wild populations (Fig. 2B and C). This divergence likely reflects a history shaped by multiple glacial refugia during the Last Glacial Maximum, followed by postglacial colonization through long-distance dispersal. Geographical isolation, combined with prolonged selfing under the unique ecological conditions of Yili, may have reinforced this genetic differentiation—an example of geographical parthenogenesis. The distinct evolutionary trajectory of the Yili population is further supported by reduced nucleotide diversity and a high proportion of pure sites (Fig. 2C).

In contrast, our data reveal that ancient landraces and cultivated walnuts from other regions of Xinjiang are closely related to wild walnuts from the Middle East, suggesting a shared origin (Fig. 2A–C and Fig. S6). This finding challenges earlier hypotheses that Xinjiang walnuts primarily originated from Wild-Yili_China—a view based on morphological characteristics [5] and early marker analyses using SSRs, AFLPs, and RAPDs [34, 35]. Further analyses suggest frequent introgression between Middle Eastern wild walnuts and ancient landraces in Hotan. From Hotan, one lineage likely spread northeast along the Tianshan Basin toward Kashi and Aksu, while another extended westward into Central Asia. The spread of these walnut lineages may have accompanied major commercial exchange activities, given that Hotan is an important historical trade hub connecting the Middle East and China [4, 36, 37]. Additionally, cultural exchanges—particularly those associated with ancient language phyla—may have played a pivotal role in the spread of walnut germplasm, as suggested by Pollegioni *et al.* [4]. Together, these findings highlight the distinct evolutionary trajectories and domestication histories of the Wild-Yili_China and Ancient Landrace-Xinjiang_China groups, reflecting their adaptation to different environmental conditions and selection pressures. Understanding these genetic distinctions is crucial for conservation strategies and future breeding programs aimed at enhancing walnut quality and resilience.

### Analysis of deleterious mutations reveals walnut adaptation to environmental stresses

The accumulation of deleterious mutations poses a significant challenge in crop improvement, as even weakly deleterious variants can persist and reduce overall fitness. Recent advances in sequencing technologies have enabled the identification of such variants in walnut, allowing us to explore their distribution, characteristics, and functional implications across different walnut populations. Our findings offer important insights into how deleterious mutations interact with domestication and adaptation processes (Fig. S7).

First, we observed that domestication has led to an increase in heterozygous deleterious mutations in the genomes of ancient landrace walnuts, supporting the traditional ‘cost of domestication’ hypothesis. This suggests that during domestication, artificial selection prioritized agronomic traits over purging genetic load, inadvertently allowing deleterious variants to accumulate. In contrast, the Wild-Yili_China population exhibited a reduced genetic load, likely due to an elevated inbreeding rate that facilitates more efficient removal of deleterious alleles through purifying selection. This observation aligns with studies in other perennial and clonally propagated crops, such as grape [26] and cassava [38], but diverges from findings in annual crops like soybean [39] and sorghum [40]. These differences likely reflect variations in crop life cycle, reproductive modes, and ecological adaptability, underscoring the importance of considering specific genetic backgrounds and environmental conditions when analyzing deleterious mutations across diverse crop systems.

Second, our analysis revealed an enrichment of deleterious mutations in genomic regions under positive selection, indicative of a hitchhiking effect, in which beneficial alleles favored during domestication dragged linked deleterious variants to higher frequencies. This phenomenon likely contributed to the genetic differentiation observed between the Wild-Yili_China and Ancient Landrace-Xinjiang_China groups. Following this differentiation, populations were further shaped by selection for stress adaptation, driving the evolution to cope with diverse environmental conditions. Consequently, eliminating deleterious variants should be a key priority in walnut breeding programs to improve stress tolerance.

Finally, we found no significant association between deleterious variants and key quality traits such as oil and tannin content. This suggests that the accumulation of deleterious variants during domestication may not have substantially impacted the metabolic pathways governing oil and tannin production. Instead, deleterious variants appear to play a more prominent role in stress adaptation, as evidenced by their distribution and evolutionary patterns within the populations studied. Alternatively, the lack of association might be attributed to the genetic structure of our study population, which predominantly comprises ancient landraces. In this population, nearly 80% of deleterious variants are rare (low frequency), which presents significant challenges for association analysis, especially when using conventional statistical models, potentially masking subtle associations with complex quality traits.

### Identification of key genes/loci lays the foundation for walnut genetic improvement

In this study, we identified significant genomic regions contributing to the differentiation between Wild-Yili_China and Ancient Landrace-Xinjiang_China groups, highlighting specific pathways crucial for walnut resilience (Table S8 and Fig. S8). Notably, genes enriched in the RIG-I-like receptor, NOD-like receptor, and MAPK signaling pathways were implicated in biotic and abiotic stress responses, underscoring their roles in walnut adaptation across diverse environments. Furthermore, several cytochrome P450 family genes involved in isoflavonoid biosynthesis, previously linked to drought stress tolerance [22], were identified as potential targets for improving walnut stress resilience.

Our GWAS analysis yielded significant new insights into the candidate genes of important walnut kernel traits (Table S8). Additional candidate loci and genes regulating tannin and oleic acid content in walnut kernels were identified. Specifically, we identified a key SNP (Jr7D-7826622) associated with two UDP-glycosyl transferases that catalyze the conversion of gallic acid to β-glucogallin, a crucial step in hydrolysable tannin biosynthesis [28]. Moreover, genes encoding an E3 ubiquitin-protein ligase and those belonging to the ABC transporter family were identified to potentially regulate lipid biosynthesis and facilitate oleic acid accumulation. In addition to nutritional profiles, our study also elucidated the genetic basis of walnut shell thickness. We identified *JrCYP98A2*, a gene in the lignin biosynthesis pathway, as a key regulator of secondary cell wall development. Overexpression of *JrCYP98A2* led to enhanced lignin production and increased levels of phenolic compounds in the phenylpropanoid pathway, resulting in thicker cell walls and greater lignification—traits directly contributing to increased shell thickness, which is important for postharvest processing and market value. Our study provides a comprehensive understanding of the genetic mechanisms underlying these important walnut traits, laying crucial groundwork for targeted breeding strategies aimed at enhancing both the nutritional profile and stress resilience of future walnut cultivars.

In summary, this study redefines the genetic landscape of Xinjiang walnuts, revealing two independent centers of diversity and challenging the established hypothesis that Xinjiang’s landraces originated primarily from the Wild-Yili_China population. We demonstrate that the Wild-Yili_China population constitutes a distinct genetic pool, shaped by postglacial isolation and efficient purifying selection, resulting in a low genetic load (Fig. S6). In contrast, Southern Xinjiang ancient landraces, which show a higher ‘cost of domestication’, are genetically allied with Middle Eastern populations (Fig. S7). This divergence was primarily driven by adaptation to environmental stress. Furthermore, by integrating GWAS and functional analysis, we identified key candidate genes regulating tannin content, oleic acid accumulation, and shell thickness (Fig. S8). These findings identify the Yili wild germplasm as a crucial, untapped resource for future walnut breeding.

## Materials and methods

### Sample collection and phenotype evaluation

A total of 75 wild accessions and 193 ancient landraces were collected from 19 locations across 17 counties in five regions of southern Xinjiang, including Kashgar, Hotan, Aksu, Bayingolin Mongol Autonomous Prefecture, and Yili Kazakh Autonomous Prefecture. These regions cover the entire oasis zone of the Tarim Basin, where ancient walnut trees are naturally distributed. Specifically, wild walnut germplasm was collected from the Ketmen Mountains Wild Walnut Nature Reserve in Gongliu County, Yili Kazakh Autonomous Prefecture (43°19′56″N–43°23′40″N, 82°13′28″E–82°16′23″E). Additionally, the study included 11 modern cultivars developed in Xinjiang and three outgroup species (*J. nigra*, *J. hopeiensis*, and *C. illinoinensis*). Fresh leaf samples were collected, immediately frozen in liquid nitrogen, and stored at −80°C for subsequent analyses.

Phenotypic measurements were performed using 10 mature walnuts per sample, which were naturally air-dried, with three biological replicates. Tannin content in walnut kernels was determined using the spectrophotometric method [41], while the composition and relative abundance of fatty acids were analyzed using the fatty acid methyl esterification method [42]. Shell thickness was measured using an electronic Vernier caliper with a precision of 0.01 mm.

### DNA library construction and sequencing

Genomic DNA was extracted from leaf samples using the Plant Polysaccharide and Polyphenol Genomic DNA Extraction Kit (Tiangen, Beijing, China), following the manufacturer’s protocol. DNA concentration was measured using a NanoDrop 2000 spectrophotometer (NanoDrop Technologies, Wilmington, DE, USA), and DNA integrity was evaluated by 1% agarose gel electrophoresis. Genomic DNA was subsequently fragmented using a Covaris ultrasonicator (Covaris, East Sussex, UK).

DNA libraries were prepared using the TruSeq Library Construction Kit (Illumina, San Diego, CA, USA) and quantified with a Qubit 2.0 fluorometer (Thermo Fisher Scientific, Waltham, MA, USA). Library fragment size distribution was assessed using an Agilent 2100 Bioanalyzer (Agilent Technologies, Santa Clara, CA, USA). Following adaptor ligation, libraries were linearly amplified via LM-PCR, denatured into single strands, and circularized to generate DNA nanoballs through rolling circle amplification. Sequencing was performed on the BGI DNBSEQ-500 platform. Additional sequencing data for other accessions were obtained from our previous study [20].

### Identification of genomic variants

Raw genomic sequencing data from 282 accessions were processed to remove adapter sequences, as well as reads containing more than 10% unidentified nucleotides (N) or with greater than 50% low-quality bases. The resulting high-quality clean reads were aligned to the reference walnut genome using BWA-MEM (version 0.7.17-r1188) with parameters ‘-t 4 -M -R’ [43]. Following alignment, SAM files were converted to BAM format and sorted by genomic coordinates using SAMtools (v1.9) [44]. SNPs and InDels were identified using the UnifiedGenotyper module in the Genome Analysis Toolkit (GATK v3.4) [45]. To ensure high-confidence variant calling, SNPs and InDels were subjected to comprehensive quality filtering. Loci were excluded if their mapping depths were less than one-third or greater than twice the average sequencing depth, if they were non-biallelic sites, or if they had missing genotype information. Genomic distribution of SNPs and InDels was visualized using shinyCircos-V2.0 [46].

### Population genetic analysis

A phylogenetic tree was constructed using the neighbor-joining (NJ) method and visualized using iTOL (https://itol.embl.de/). PCA was performed using EIGENSOFT (v.7.2.1) [47], with the input PED file generated using PLINK (http://pngu.mgh.harvard.edu/~purcell/plink/). Population structure was inferred using FRAPPE (http://med.stanford.edu/tanglab/software/frappe.html) based on ancestry proportion estimation. LD, measured as *r*^2^ between SNP pairs, was calculated using TASSEL (v.5.0) [48], and LD decay patterns were analyzed using PopLDdecay (v.3.30, https://github.com/BGI-shenzhen/PopLDdecay) [49]. The kinship matrix among accessions was estimated using the GAPIT package (v3.0) [50].

### Genetic diversity and population differentiation

Genetic diversity was assessed by estimating the number of alleles (NA), observed heterozygosity (Ho), and unbiased expected heterozygosity (He) using Genepop (v.1.2) [51]. Pairwise *F*_ST_ values were calculated to assess population differentiation using Genepop.

### Population history and gene flow analysis

Population demographic history was inferred using PSMC (v.0.6.5) [52], as previously reported [20]. To estimate gene flow between wild walnut accessions and ancient landraces, Treemix (v.1.12) was employed to calculate effective migration rates per generation. Migration analysis was conducted using a maximum likelihood approach to estimate migration rates and effective population sizes simultaneously. Gene flow was further assessed by examining ABBA-BABA test statistics, with D-statistics computed using the qpDstat command in the AdmixTools package [53]. Wild walnut samples from southwestern China were used as the outgroup in all four-population tests. Gene flow was considered statistically significant when the absolute *Z*-score exceeded 3 (|*Z*| > 3).

### IBD segments among subpopulations

IBD regions between pairs of accessions were identified using nonoverlapping 10-kb sliding windows, with each window containing a minimum of 10 SNPs. Similarity scores were calculated using the p-distance method, and windows with the top 5% of similarity scores were considered as IBD regions. The percentage of the genome covered by IBD windows was calculated for each pair of accessions. The mean percentage of IBD across all sliding windows was computed following the approach described in a previous study [11].

### Selective sweep and GWAS analysis

Selective sweep analysis was performed using VCFtools (v.0.1.13) [54] to calculate *F*_ST_ and ROD values across 100-kb sliding windows with a 10-kb step size between subpopulations. GWAS for tannin and oleic acid content were conducted using the FarmCPU model, which incorporates random effects to account for kinship and population structure, as implemented in GAPIT (v.3.0) [55].

### Identification of deleterious mutations

Deleterious mutations were identified from nonsynonymous SNP sites annotated using SnpEff [56]. SNPs with a SIFT score <0.05, as calculated by the SIFT software [57], were considered potential deleterious mutations. Candidate deleterious mutations were also identified using PROVEAN [58]. Only SNPs predicted as deleterious by both SIFT and PROVEAN were considered reliable deleterious mutations for downstream analysis.

### Transcriptome sequencing and data analysis

Total RNA was extracted using the EASYspin Total RNA Extraction Kit (Biomed, Beijing, China). RNA integrity was assessed using 1.2% agarose gel electrophoresis and a NanoPhotometer® spectrophotometer (IMPLEN, CA, USA). RNA-Seq libraries were constructed and sequenced on the Illumina platform. Low-quality reads and adaptor sequences were removed to obtain high-quality clean data.

Differential expression analysis was performed using DESeq2 (v.1.6.16.1) [59]. Genes with a fold change ≥2 and a false discovery rate <0.01 were considered differentially expressed. Gene expression levels were quantified as FPKM (fragments per kilobase of transcript per million mapped reads). GO annotations were obtained from the InterPro database, and KEGG pathways were annotated using the KEGG database (http://www.genome.jp/kegg/kaas/).

### Subcellular localization of JrCYP98A2

The CDS of *JrCYP98A2* was cloned into the pC2300-GFP expression vector, which was subsequently introduced into *Agrobacterium tumefaciens* strain GV3101 via electroporation. Transformants were cultured on selective medium at 30°C for 2 days. Positive clones were incubated to an OD600 of ~0.6, then harvested by centrifugation at 4000 rpm for 4 min. The cell pellet was resuspended in 10 mM MgCl₂ supplemented with 120 μM acetosyringone. Approximately 1 ml of the resulting suspension was infiltrated into the lower epidermis of leaves from healthy tobacco plants using a needleless syringe. Plants were maintained under low light conditions for 2 days following infiltration. GFP fluorescence in infiltrated tobacco leaves was examined using a laser confocal microscope.

### Genetic transformation and lignin monomer analysis

The CDS of *JrCYP98A2* was cloned into the PHB vector to generate the 35S::*JrCYP98A2* overexpression construct. The recombinant plasmid was introduced into *A. tumefaciens* strain GV3101 and subsequently transformed into *A. thaliana* (Col-0) using the floral dip method. Seeds from the transformed plants were germinated on half-strength Murashige and Skoog (1/2 MS) medium containing 0.7% (w/v) agar, 1% (w/v) sucrose, and kanamycin (50 mg/l). Wild-type plants and homozygous overexpression lines were selected and grown under controlled light conditions. Upon maturation, leaves, siliques, roots, and stalks were collected for lignin monomer analysis.

The composition and relative abundance of lignin monomers were analyzed using gas chromatography–mass spectrometry. The microstructures of *A. thaliana* tissues were observed using an SEM, and additional anatomical features were visualized by toluidine blue staining. Quantification of cell wall thickness in stem xylem cells, as well as measurement of lignified area ratios in cross-sections of *Arabidopsis* siliques and stems, was performed using ImageJ [60].

## Supplementary Material

Web_Material_uhag082

## Data Availability

Whole-genome resequencing data of 282 walnut accessions have been deposited at the National Genomics Data Center (https://ngdc.cncb.ac.cn/) with the accession code CRA028251.
