# Supplementary material for: Genomic divergence, adaptation, and the genetic basis of quality traits in ancient walnut landraces and wild relatives
Source: Hortic Res. 2026 Mar 31;13(7):uhag082. doi: 10.1093/hr/uhag082 (PMC13322160; doi:10.1093/hr/uhag082)
Supplement: Web_Material_uhag082 [file web_material_uhag082.zip › Supplementary_figures.docx]

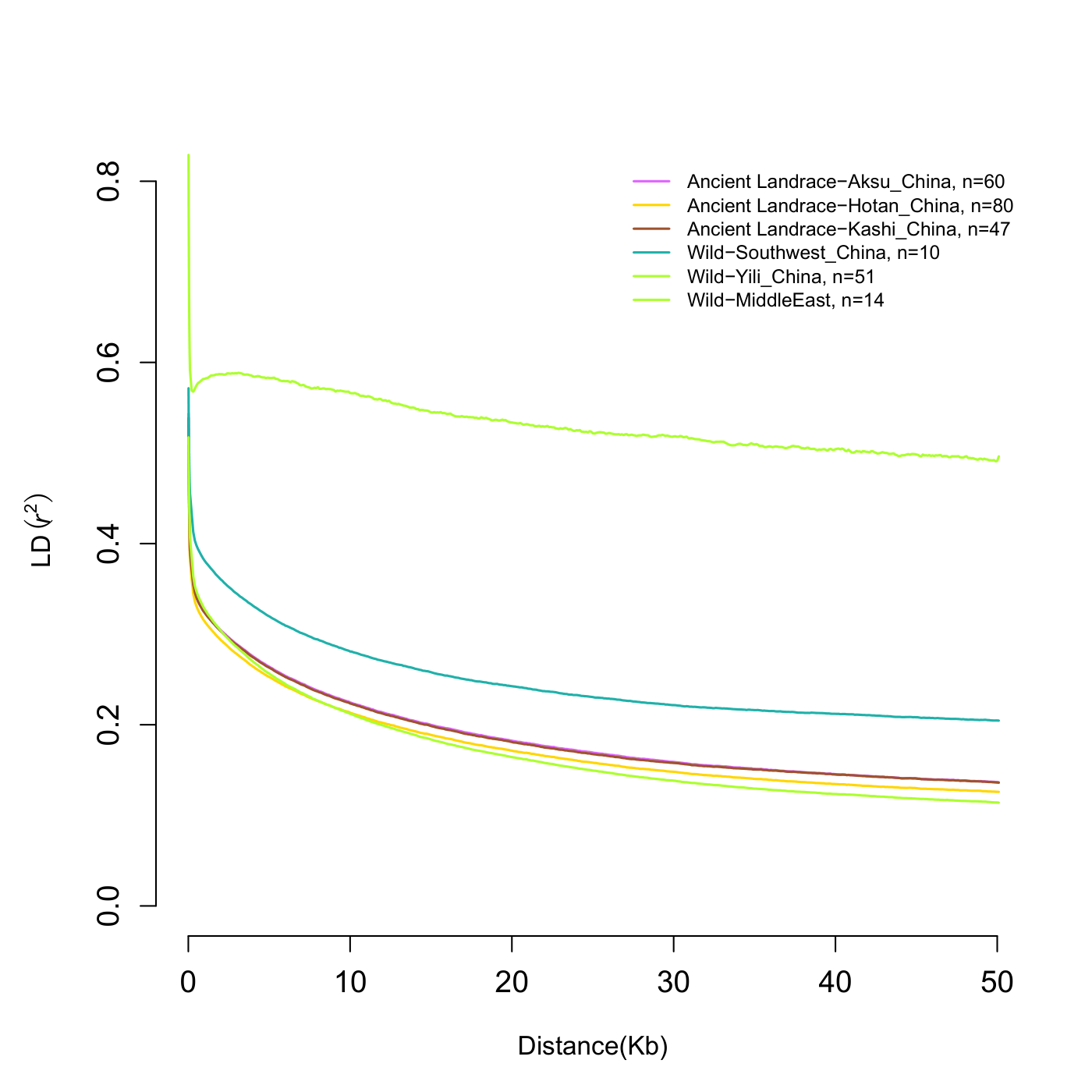


**Figure S1. Linkage disequilibrium (LD) analysis of different walnut subpopulations.**


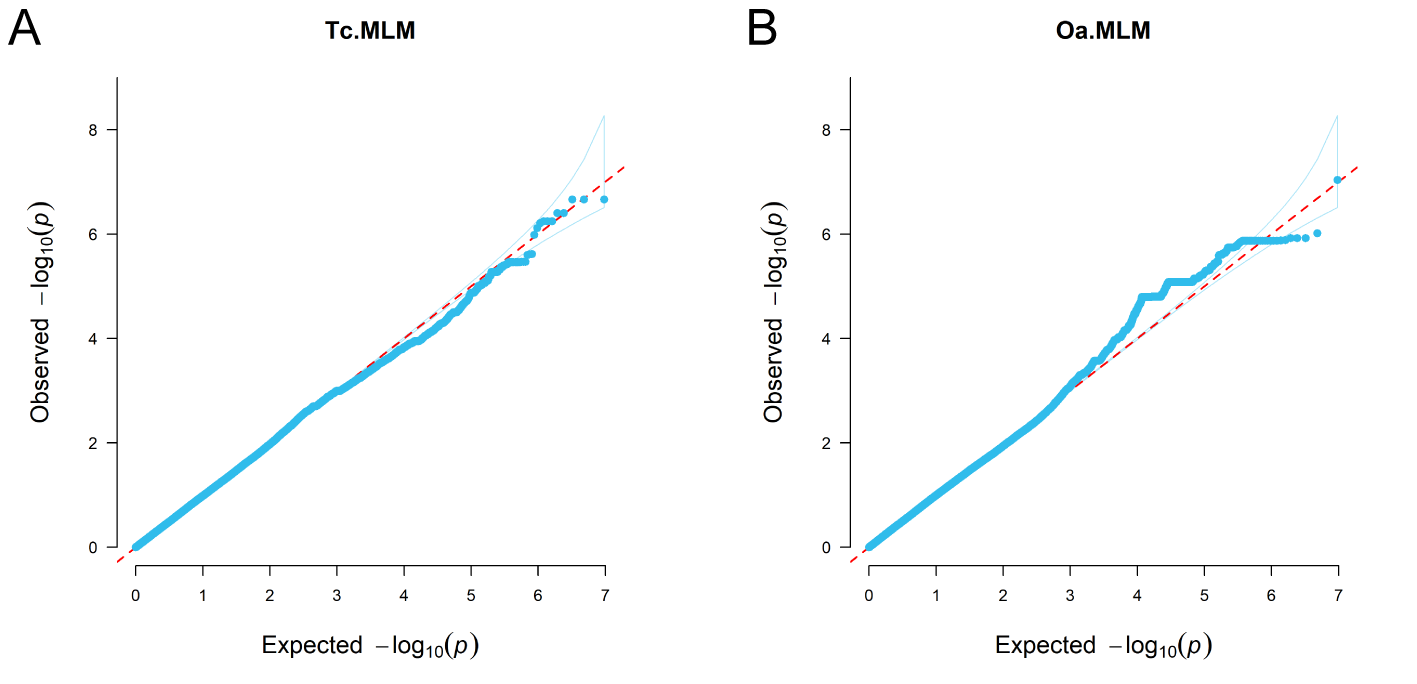


**Figure S2. QQ-plot for tannin (A) and** **oleic acid (B) content in walnut.**


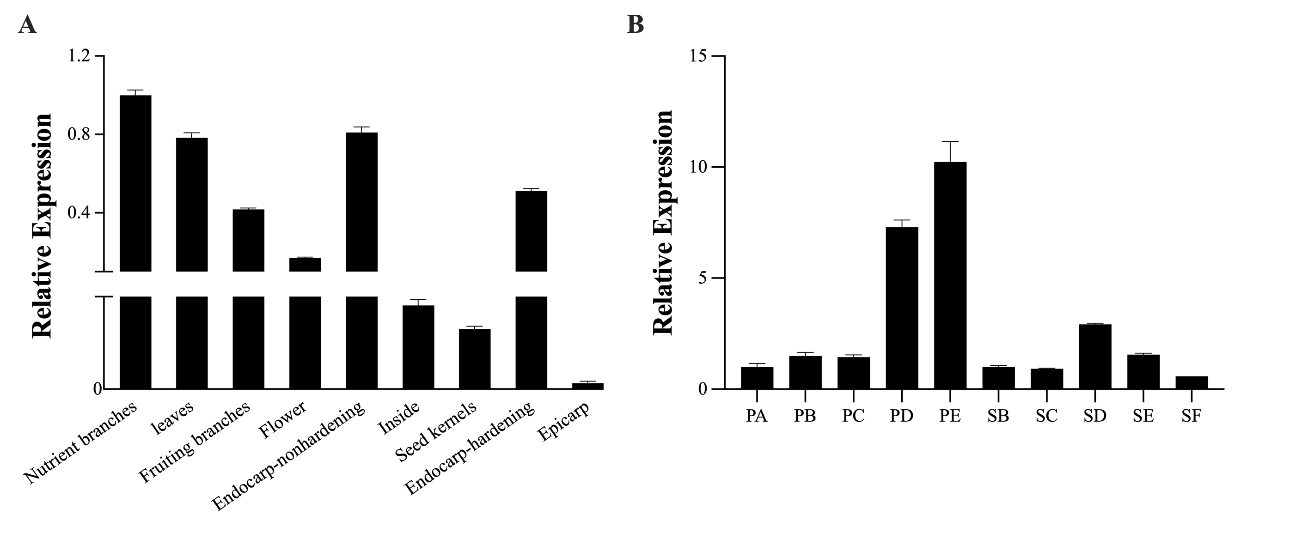


**Figure S3. Expression profile of *JrCYP98A2* across various tissues and developmental stages in walnut*.***

(A) Expression levels of *JrCYP98A2* in different walnut tissues. (B) Expression levels of *JrCYP98A2* at different kernel developmental stages. PA (P for Pulpy hue), endocarp collected at 50 days after full bloom (DAFB) (June 6). PB, non-lignified samples collected at 64 DAFB (June 20). SB (S for Scleritic hue), lignified samples collected at 64 DAFB (June 20). PC, non-lignified samples collected at 71 DAFB (June 27). SC, lignified samples collected at 71 DAFB (June 27). PD, non-lignified samples collected at 78 DAFB (July 4). SD, lignified samples collected at 78 DAFB (July 4). PE, non-lignified samples collected at 92 DAFB (July 18). SE, lignified samples collected at 92 DAFB (July 18). SF, lignified endocarp samples collected at 99 DAFB (July 25).


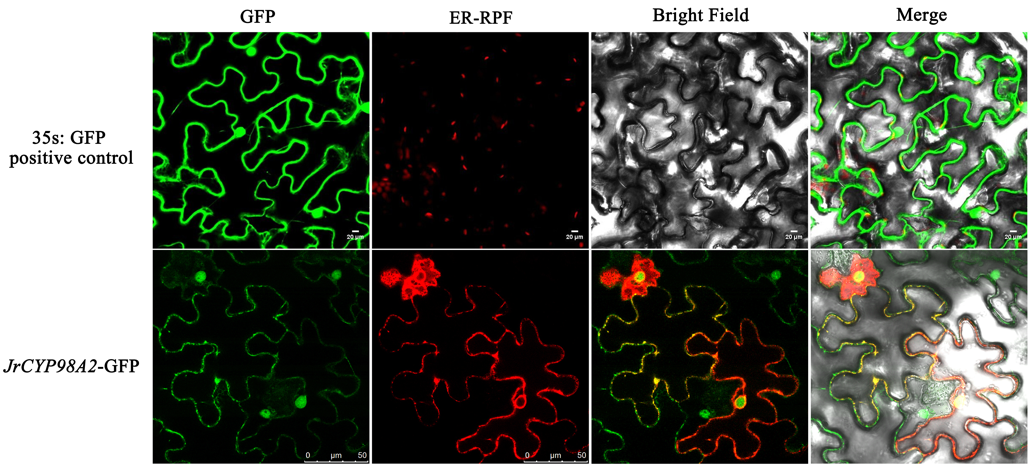


**Figure S4. Subcellular localization of the JrCYP98A2 protein in Walnut.**


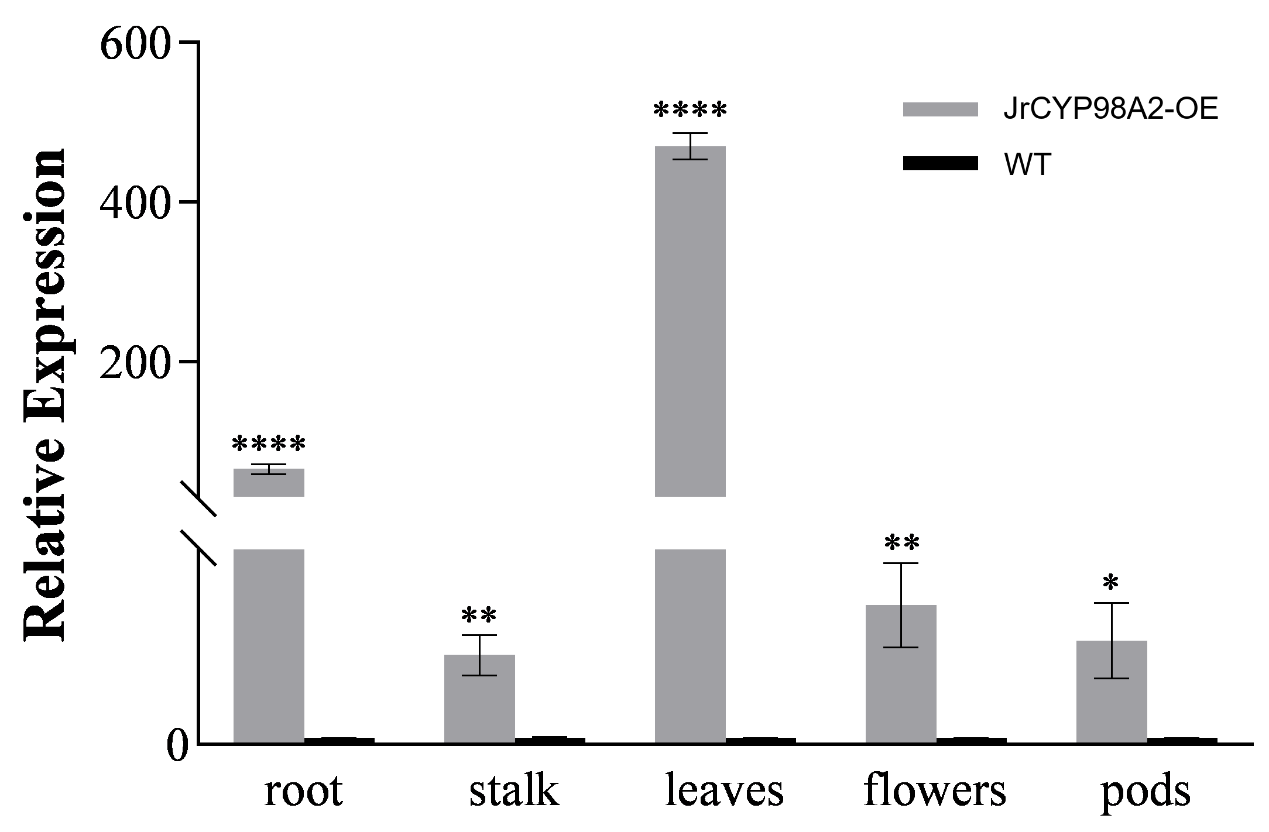


**Figure S5. Expression levels of *JrCYP98A2* in transgenic *Arabidopsis thaliana* plants compared with wild-type (WT) controls.**

****, significance difference at *P* < 0.0001; **, *P* < 0.01; *, *P* < 0.05.


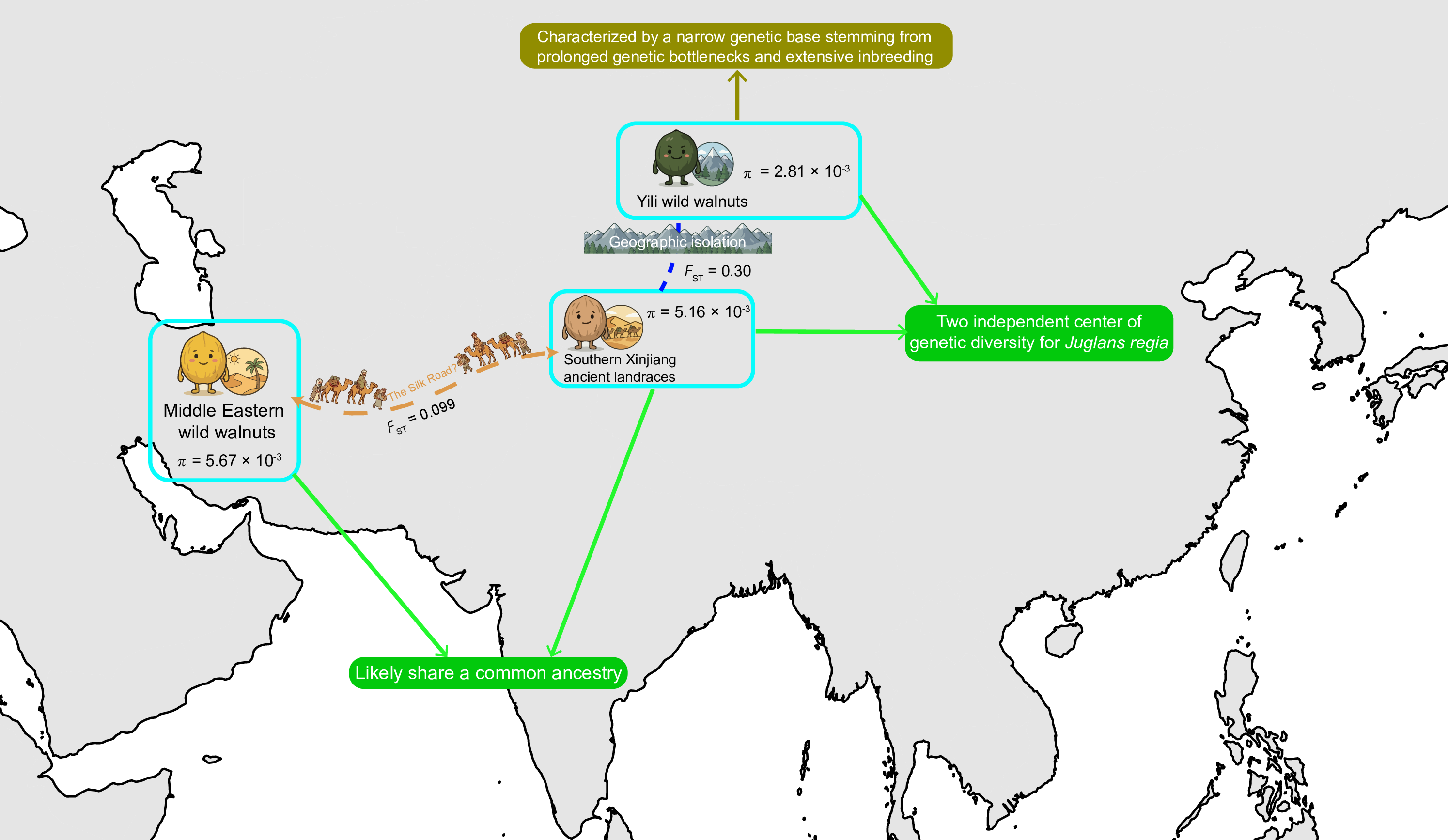


**Figure S6. Schematic representation of the dual origins and genetic distinctiveness of Xinjiang walnuts.**

The diagram illustrates the divergence between the wild populations in Northern Xinjiang (Yili) and the ancient landraces in Southern Xinjiang. The Yili wild walnuts constitute a distinct, isolated gene pool shaped by natural selection and genetic drift (bottlenecks), featuring lower diversity. In contrast, the Southern Xinjiang ancient landraces are genetically distinct from the Yili population but closely related to Middle Eastern wild walnuts. This supports the hypothesis that Southern Xinjiang landraces did not originate from local wild populations but were likely introduced from the Middle East through human-mediated dispersal along the Silk Road, contributing to their higher genetic diversity compared to the isolated wild lineages.


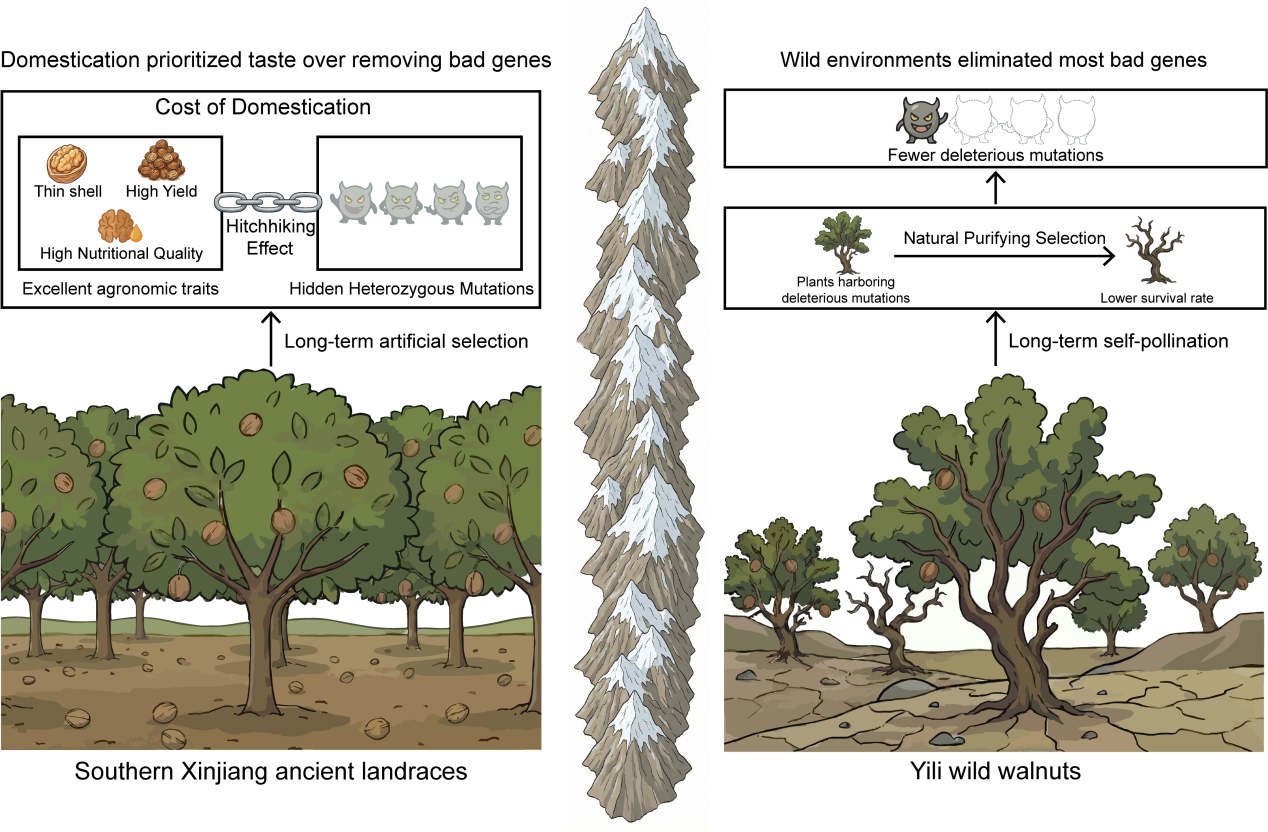


**Figure S7. Contrasting evolutionary forces shaping the landscape of deleterious mutations in Xinjiang walnuts.**

The schematic illustrates the divergent accumulation patterns of deleterious mutations between Southern Xinjiang ancient landraces (left) and Yili wild walnuts (right). (Left) In ancient landraces, long-term artificial selection prioritized beneficial agronomic traits (e.g., thin shell, high yield). However, owing to the "hitchhiking effect", deleterious mutations (represented by translucent "imps") linked to these favorable alleles were inadvertently retained and accumulated in a heterozygous state, a phenomenon known as the "cost of domestication". (Right) In contrast, the Yili wild population, subject to harsh environmental pressures and long-term self-pollination, underwent rigorous natural purifying selection. Individuals harboring high genetic loads exhibited lower survival rates, leading to the efficient purging of deleterious alleles and a cleaner genetic background.


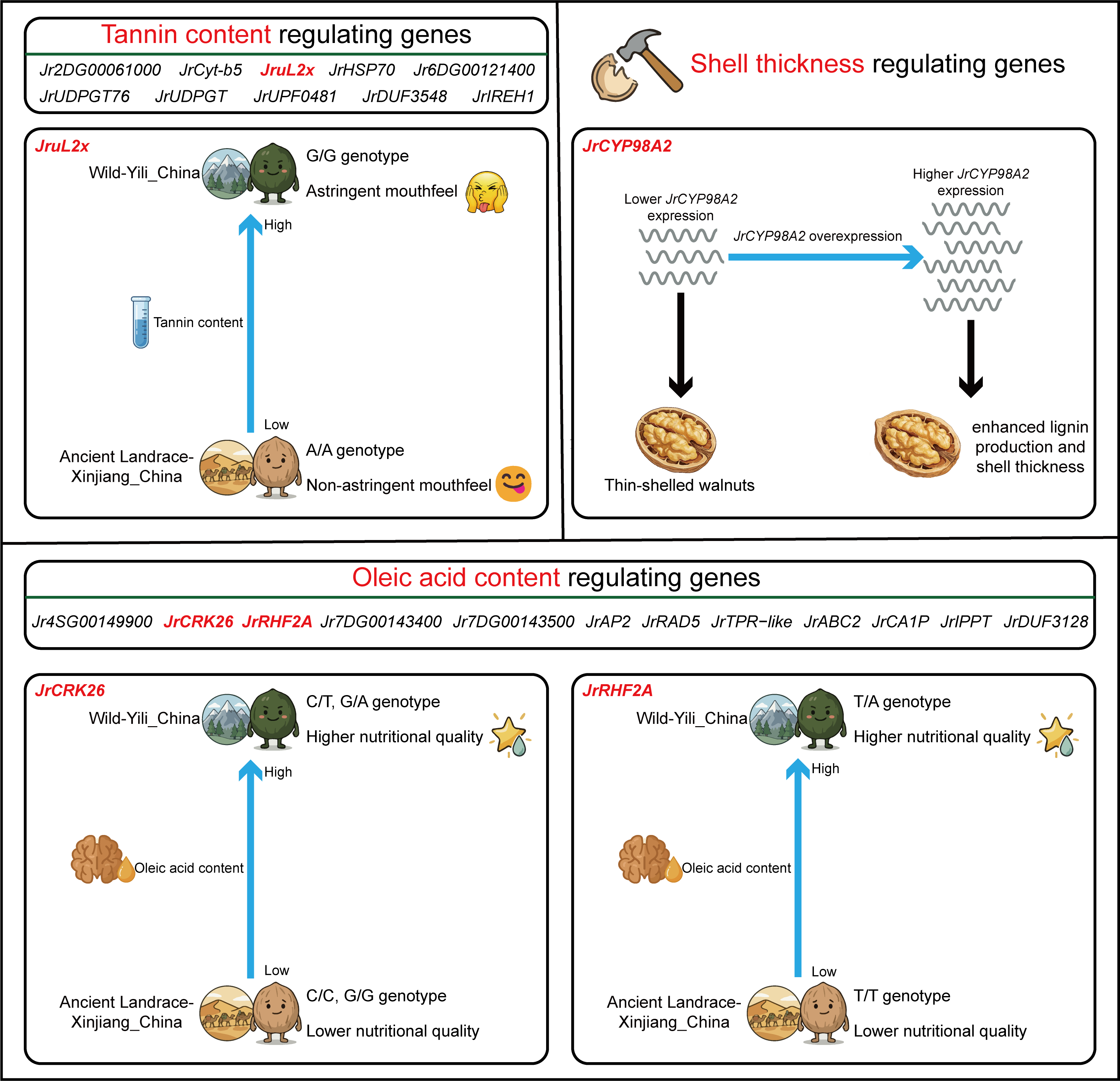


**Figure S8. Genetic architecture and candidate genes regulating key quality traits in walnut kernels.**

The diagram summarizes the key loci and molecular mechanisms underlying three economically important traits: **(Top Left) Tannin Content:** GWAS identified 10 candidate genes, including *JruL2x*. Accessions carrying the A/A genotype at the *JruL2x* locus exhibit significantly lower tannin content and a non-astringent mouthfeel, whereas the G/G genotype is associated with high astringency. **(Top Right) Shell Thickness:** The gene *JrCYP98A2* was identified as a key regulator of lignification. Overexpression of *JrCYP98A2* enhances lignin biosynthesis, leading to thicker shells, while lower expression is associated with the desirable thin-shelled phenotype. **(Bottom) Oleic Acid Content:** GWAS revealed 12 candidate genes, with *JrCRK26* and *JrRHF2A* playing pivotal roles. For *JrCRK26*, the Geno2 (C/T, G/A) genotype is linked to higher oleic acid accumulation and superior nutritional quality, a trait notably enriched in Yili wild walnuts. Similarly, the T/A genotype of *JrRHF2A* is associated with elevated oleic acid levels compared to the T/T genotype.
